# GCN2 controls the cellular checkpoint: potential target for regulating inflammation

**DOI:** 10.1038/s41420-017-0022-5

**Published:** 2018-02-14

**Authors:** Xiaojing Xia, Liancheng Lei, Wanhai Qin, Lei Wang, Gaiping Zhang, Jianhe Hu

**Affiliations:** 1 College of Animal Science and Veterinary Medicine, Henan Institute of Science and Technology, Xinxiang, China; 2 Postdoctoral Research Base, Henan Institute of Science and Technology, Xinxiang, China; 3 Post-doctoral Research Station, Henan Agriculture University, Zhengzhou, China; 4 College of Veterinary Medicine, Jilin University, Changchun, China; 5 Center for Experimental and Molecular Medicine, Academic Medical Center, University of Amsterdam, Amsterdam, The Netherlands

**Fig. 1:**
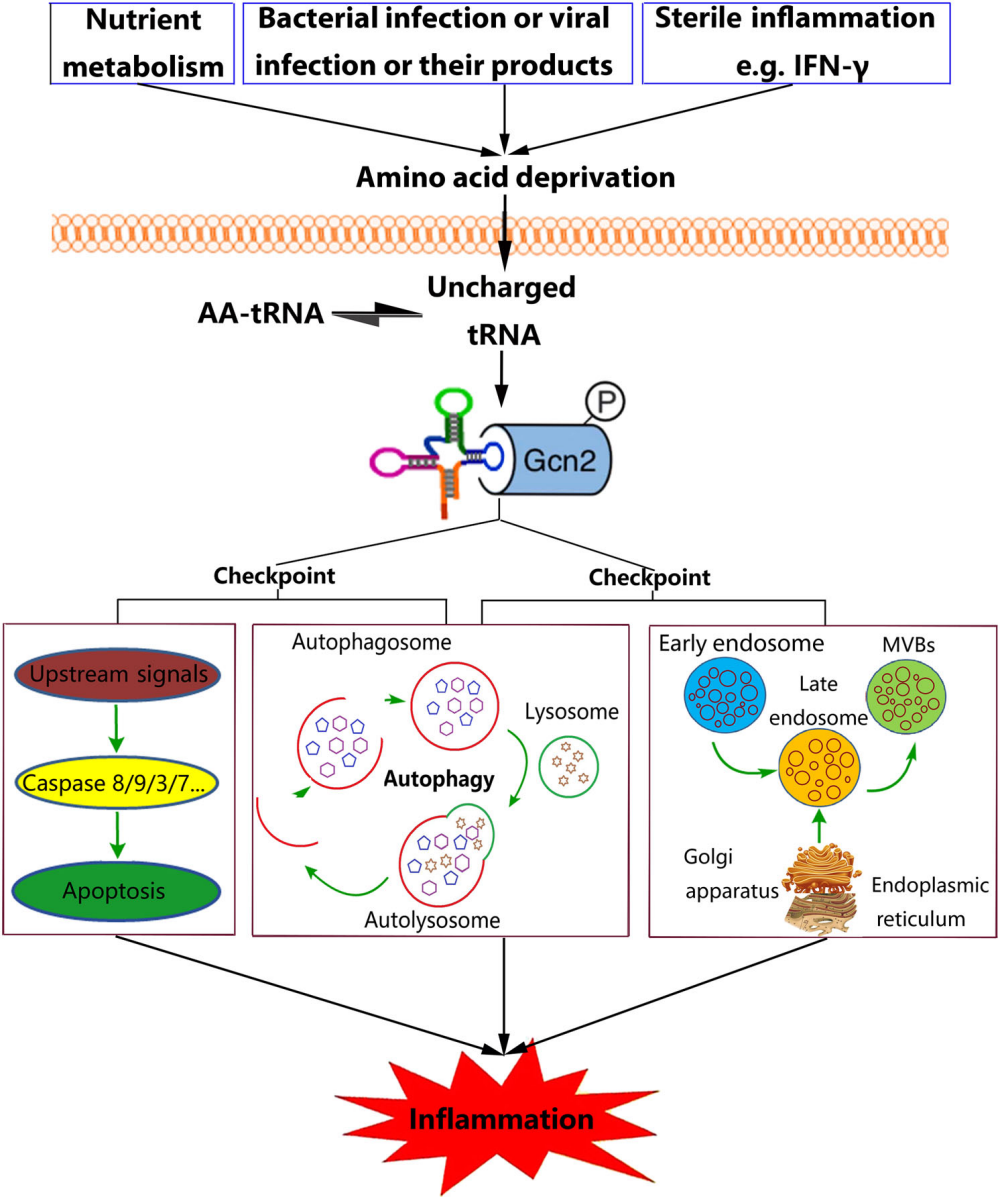
The checkpoint role of GCN2 during inflammation. Different conditions can stimulate amino acid deprivation and increase levels of uncharged tRNA, which activate the amino acid sensor GCN2. This represents a checkpoint whereby cells irrevocably commit to apoptosis, autophagy or the release of exosomes, which may determine the outcome of the inflammatory response

Accumulating evidence suggests that cellular stress signals, including those induced by nutrient availability, strongly influence the function of the immune system. The integrated stress response (ISR) is a cytoprotective response mediated by eukaryotic translation initiation factor 2α (eIF2α) that enables cells to sense and respond to diverse cellular signals, including endoplasmic reticulum (ER) stress, the unfolded protein response and nutrient deprivation. GCN2, one of four known sensors of the ISR, can be activated by single amino acid deprivation. In the context of low levels of an amino acid, uncharged tRNA molecules accumulate and bind to the HisRS domain of GCN2 (Fig. 1). Subsequently, the phosphorylation of eIF2αin Ser-51 occurs, which inhibits its function, leading to reduced mRNA translation and protein synthesis, thereby reducing the amino acid supply. Concurrently, eIF2α phosphorylation enhances the translation of specific mRNA molecules that contain 5ʹ-terminal leader sequences. These molecules include the transcription factor ATF4, which controls the transcription and expression of hundreds of genes, maintains cell homeostasis, and participates in protein metabolism, host responses to infection, responses to immunization, inflammation and other physiological and pathological processes^1^. As nutrients are metabolized, dynamic changes in nutrient bioavailability occur in the intestine, which can trigger metabolic sensors such as GCN2 and potentially modulate gut immune responses. In a recent report published in *Nature*, Ravindran et al. demonstrated that GCN2-mediated amino acid starvation-sensing mechanisms can shape intestinal inflammation in the dextran sodium sulfate (DSS) mouse model of colitis, indicating that GCN2 has the ability to modulate immune responses, especially inflammation (Fig. 1)^2^.

In our previous study, we showed another setting in which GCN2 is activated and IFN-gamma (IFN-γ) accelerates bovine mammary epithelial cell (BMEC) arginine consumption, resulting in activation of the amino acid starvation response that further drives GCN2 activity in a lactating Holstein cow model, suggesting that GCN2 can be activated by non-infectious (i.e., sterile) inflammation (Fig. 1)^3^. During infection, bacterial or viral growth may cause a nutrient shortage in cells or tissues, which might activate GCN2 signaling (Fig. 1)^4,5^. Damage of the plasma membrane or inhibition of amino acid uptake by pore-forming toxins (PFT), such as α-toxin, also causes amino acid starvation and energy loss, resulting in GCN2 activation (Fig. 1)^4^. Thus, nutrient and energy sensors such as GCN2 may serve as sentinels for the initiation of immune responses.

In the experimental autoimmune encephalomyelitis mouse model, central nervous system inflammation was enhanced after GCN2 deletion, which was characterized by increased expression of IL-17 and IFN-γ and decreased expression of IL-10 during the remission phase, resulting in enhanced nervous tissue inflammation and demyelinating lesions^6^. Activation of GCN2 in macrophages promotes the expression of anti-inflammatory cytokine IL-10 in vitro. In vivo, apoptotic cells can stimulate the production of anti-inflammatory cytokines IL-10 and TGF-β in macrophage in a GCN2-dependent manner, whereas myeloid cell-specific deletion of GCN2 abrogated regulatory cytokine production that resulted in increased immune cell activation, humoural autoimmunity, renal pathology, and mortality^7^. In the lupus nephritis mouse model, a GCN2 agonist significantly reduced the release of inflammatory cytokines and decreased mortality in mice^8^. Thus, GCN2 have a widely distribution in various system and are extensively involved in inflammatory responses.

Following the activation of GCN2, a series of events occur that prevent gut inflammation^2^. In addition to the phosphorylation of eIF2α by GCN2, other mechanisms contribute to gut inflammation. Indeed, defective autophagy was found to mediate enhanced gut inflammation in GCN2-deficient mice. Autophagy is an evolutionarily conserved catabolic process in which cytoplasmic proteins and organelles are directed to lysosomes for degradation and recycling. It is an essential process for maintaining cellular homeostasis and cell survival during inflammation. Autophagy also participates in the regulation of inflammation, as conditional ablation of components of the autophagic machinery results in higher levels of oxidative stress and inflammasome activation. Inflammasome activation results in increased production of the proinflammatory cytokines interleukin (IL)-1β and IL-18^2^. In contrast to pro-survival pathways, which include autophagy, pro-death processes, such as apoptosis, also play important roles in inflammation as regulating cell death can both remove damaged cells to contribute to the restoration of normal tissue or organ structure and function and limit the spread of micro-organisms via direct killing or deprivation of cellular materials needed for micro-organism survival and replication^9^. Interestingly, in another study, the Muaddi Group reported a pro-apoptotic role for GCN2^10^, indicating that GCN2 can have both pro-survival and pro-death activities. Cell death is a major determinant of the severity of inflammation. The pro-survival and pro-death processes of autophagy and apoptosis interact and influence each other during inflammation; indeed, a checkpoint exists at which cells irrevocably commit to either pathway^9^. Whether cells undergo autophagy (live) or apoptosis (die) during inflammation may largely depend on the signals received by GCN2. Thus, GCN2 may be a critical apoptosis/autophagy checkpoint during inflammation (Fig. 1).

Exosomes represent another cellular homeostasis mechanism that, similar to autophagy, participates in the control of inflammation by promoting the release of harmful intracellular components, including proteins, lipids and nucleic acids. However, functional exosomes might not only activate the expression of target cell inflammatory factors and other inflammatory mediators by activating relevant signaling pathways but also be involved in the loading and secretion of inflammatory factors into target cells by inducing fusion with target cells and stimulating target cells to produce inflammatory factors, thereby promoting the spread of inflammation^11^. LC3 overexpression-, starvation-, or rapamycin treatment-induced autophagy can inhibit exosomes release, suggesting that under conditions that stimulate autophagy, cells are directed to the autophagic pathway with the consequent inhibition of exosome release. Thus, the balance between autophagy induction and exosome release might be regulated by the cellular metabolic state^12^. Notably, GCN2 is an important nutrient receptor. Kloft et al. showed that the GCN2 downstream target protein eIF2α regulates membrane transport, which might affect the maturation and release of exosomes^13^. Hence, GCN2 may be a critical autophagy/exosome checkpoint protein during inflammation (Fig. 1). Thus, GCN2 can have diverse functional regulatory activities in apoptosis, autophagy and exosomes. Understanding the functional diversity of GCN2 is important since the manipulation of the apoptosis/autophagy/exosome checkpoint represents a novel opportunity for the treatment of inflammatory diseases.

The concept of an apoptosis/autophagy/exosomes checkpoint has been gradually established and the latest data suggests that GCN2 is a representative of this checkpoint (Fig. 1) that can be directly regulated during inflammation. Manipulation of the checkpoint to favor cell survival or death might open up exciting new therapeutic options for a number of various chronic inflammatory diseases. For example, an agonist of GCN2 has been identified (halofuginone), which we have established to inhibit LPS-induced inflammation in dairy cow mammary epithelial cells by controlling the release of exosomes, likely because of the activation of GCN2 (unpublished observations). Proto-Siqueira et al. showed that halofuginone can induce apoptosis in mantle cell lymphoma cells via activation of GCN2^14^. Likewise, the identification and characterization of pharmacologic antagonists of GCN2 as immune activators is an active area of research that will likely yield a series of GCN2 inhibitors in the future. In addition, experimental inhibition of GCN2 signaling by amino acid supplementation can be reversed to some extent by physiological/pathological changes^4,15^. In conclusion, accumulating evidence suggests that GCN2 activity represents a compelling target and novel approaches for therapy in inflammatory diseases associated with manipulation of GCN2 will likely be developed in the future. Care must be taken, however, as the side effects and long-term effects of GCN2 signal manipulation remain unknown.
